# Vertebral disk morphology of the lumbar spine: a retrospective analysis of collagen-sensitive mapping using dual-energy computed tomography

**DOI:** 10.1007/s00256-020-03685-5

**Published:** 2020-12-04

**Authors:** Friederike Schömig, Matthias Pumberger, Yannick Palmowski, Ann-Kathrin Ditges, Torsten Diekhoff, Friedemann Göhler

**Affiliations:** 1 Center for Musculoskeletal Surgery, Charité – University Medicine Berlin, Charitéplatz 1, 10117 Berlin, Germany; 2 Department of Radiology, Charité – University Medicine Berlin, Charitéplatz 1, 10117 Berlin, Germany

**Keywords:** Vertebral disk, Pathology, Tomography, X-ray computed, Spine, Collagen

## Abstract

**Objectives:**

To investigate the diagnostic accuracy of collagen-sensitive maps derived from dual-energy computed tomography (DECT) for the detection of lumbar disk pathologies in a feasibility setting.

**Materials and methods:**

We retrospectively reviewed magnetic resonance imaging (MRI), computed tomography (CT), and DECT datasets acquired in patients who underwent periradicular therapy of the lumbar spine from June to December 2019. Three readers scored DECT collagen maps, conventional CT, and MRI for presence, type, and extent of disk pathology. Contingency table analyses were performed to determine diagnostic accuracy using MRI as standard of reference. Interrater agreement within and between imaging modalities was evaluated by computing intraclass correlation coefficients (ICCs) and Cohen’s kappa. Correlation between sum scores of anteroposterior disk displacement was determined by calculation of a paired *t* test.

**Results:**

In 21 disks in 13 patients, DECT had a sensitivity of 0.87 (0.60–0.98) and specificity of 1.00 (0.54–1.00) for the detection of disk pathology. Intermodality agreement for anteroposterior disk displacement was excellent for DECT (ICC 0.963 [0.909–0.985]) and superior to CT (ICC 0.876 [0.691–0.95]). For anteroposterior disk displacement, DECT also showed greater within-modality interrater agreement (ICC 0.820 [0.666–0.916]) compared with CT (ICC 0.624 [0.39–0.808]).

**Conclusion:**

Our data suggest that collagen-sensitive imaging has an added benefit, allowing more accurate evaluation of the extent of disk displacement with higher interrater reliability. Thus, DECT could provide useful diagnostic information in patients undergoing CT for other indications or with contraindications to MRI.

**Supplementary Information:**

The online version contains supplementary material available at 10.1007/s00256-020-03685-5.

## Introduction

Affecting 3–5% of the population, lumbar radiculopathy is one of the most common symptoms caused by degenerative spinal disease [1]. Patients present with pain or sensory loss in a dermatomal pattern, possibly in conjunction with motor function loss in a myotomal pattern. In most cases, the underlying cause is irritation of a specific nerve root, which in turn is most commonly caused by disk herniation or bulging, hypertrophy of the facet or ligaments, or spondylolisthesis. Radiculopathy is diagnosed by an extensive physical examination and magnetic resonance imaging (MRI), the current diagnostic standard [2].

Dual-energy computed tomography (DECT) is a fairly new CT technique but is already included in guidelines for imaging in gout [3]. More recently, it has also been introduced for the detection of bone marrow edema in the spine, e.g., in patients with vertebral fractures [4–6]. DECT uses a so-called three-material decomposition algorithm to detect calcium; the same principle can be used to identify collagenous structures such as the intervertebral disk [7].

In patients who have contraindications to diagnostic MRI or need a timely CT scan for other indications, disk visualization by CT could be helpful as an alternative diagnostic instrument. Previous studies have highlighted the value of DECT in intervertebral disk evaluation and have shown a higher sensitivity and specificity of DECT compared with standard CT in detecting lumbar disk herniation and spinal nerve root impingement [8, 9]. However, these studies used different DECT reconstruction algorithms and did not compare results with MRI—the current diagnostic standard in detecting disk pathology.

Thus, the aim of our retrospective study was to investigate the diagnostic accuracy of collagen-sensitive mapping using DECT in the detection of lumbar disk pathology in patients undergoing CT-guided lumbar spinal injection compared with standard CT and preinterventional MRI.

## Materials and methods

### Patients and ethics approval

**Fig. 1:**
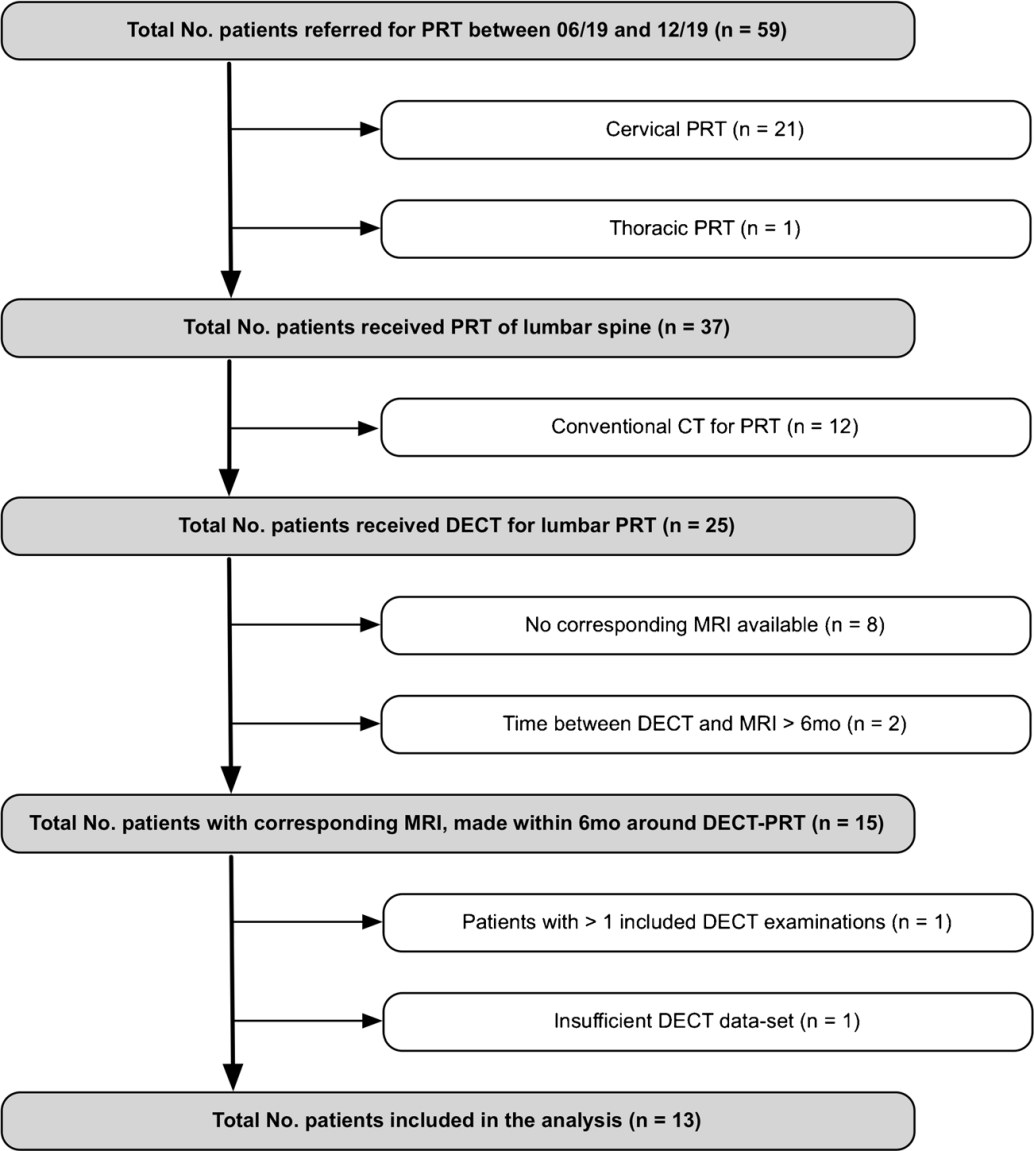
Patient flowchart. Of 25 DECT examinations performed for lumbar PRT, 13 were finally included. A total of 21 segments were scored (13 target and 8 reference levels) separately using MRI, CT, and DECT. Thirteen segments were classified positive for disk herniation using DECT and 14 using CT versus 15 using MRI

This study was approved by the local ethics committee (EA1/011/20). We retrospectively included patients who presented to our orthopedic clinic with lumbar radiculopathy and an underlying disk pathology on MRI who underwent DECT-guided lumbar spine injection in our department of radiology from June 2019 to December 2019. Inclusion criterion was availability of an axial and a sagittal T2-weighted MRI sequence. Exclusion criteria were incomplete imaging data (e.g., no MR images) and an interval of over 6 months between MRI and DECT. A flowchart of patient inclusion is shown in Fig. 1.

### Image acquisition

For the planning of lumbar spine injections, all patients underwent a DECT scan of the target region of the lumbar spine on a 320-row single-source CT scanner (Canon Aquilion ONE Vision Edition, Canon Medical Systems, Tochigi, Japan). The protocol included a scanogram and DECT with sequential volume acquisition of the high (135) and low (80) kVp datasets. Rotation time was 0.275 s with a changeover time of 0.5 s between acquisitions. Exposure control was set to a standard deviation of 12, and the so-called wide volume mode was used if necessary. The scan range was restricted to the target area and did not necessarily include the whole lumbar spine.

### Image postprocessing

Conventional CT images were computed with 0.5-mm slice thickness using iterative reconstruction (Adaptive Iterative Dose Reduction (AIDR) 3D standard) and a medium soft tissue kernel without beam hardening compensation (Filter Convolution (FC) 13) for DECT. Collagen maps were reconstructed with a primary slice thickness of 0.5 mm using a DECT raw data tool on the CT console (Dual-Energy Image View, Version 6; Canon Medical Systems) and a collagen-specific gradient of 1.10 for the three-material decomposition [10]. The corresponding MR images of the lumbar spine were from examinations the patients underwent in our department or elsewhere. Thus, MRI parameters were not standardized. Available MRI slice thicknesses ranged from 3 to 4 mm.

### Identification of target and reference disks and image postprocessing

For definition of target and reference disks, MRI datasets were screened by two musculoskeletal radiologists, defining the segments in which periradicular infiltration was performed on as target levels/disks (one level per patient). If possible, the two radiologists jointly identified one normal-appearing reference segment per patient in the same datasets.

### Anonymization

Data preparation, anonymization, and scoring were performed on a workstation with a high-resolution monitor using Horos (version 3.3.5). For this task, CT, DECT, and MR images were clipped to the segments of the respective reference or target disk and anonymized separately. In this way, the readers only had access to one modality and one vertebral segment at a time and were blinded to clinical and identifying information, findings of the other modalities and at other vertebral levels, and whether the disk was defined as target or reference.

### Scoring

Three readers (reader 1, a radiologist specializing in musculoskeletal diseases with 9 years of experience; reader 2, an orthopedic surgery resident with 1 year of experience; reader 3, a radiology resident with 1 year of experience) independently evaluated the images (CT, DECT, and MRI) on a Horos workstation. The readers used multiplanar angulated images reformatted with 3 mm slice thickness for CT and DECT and, if necessary, for T2w MR images to score sagittal and oblique axial planes.

**Fig. 2:**
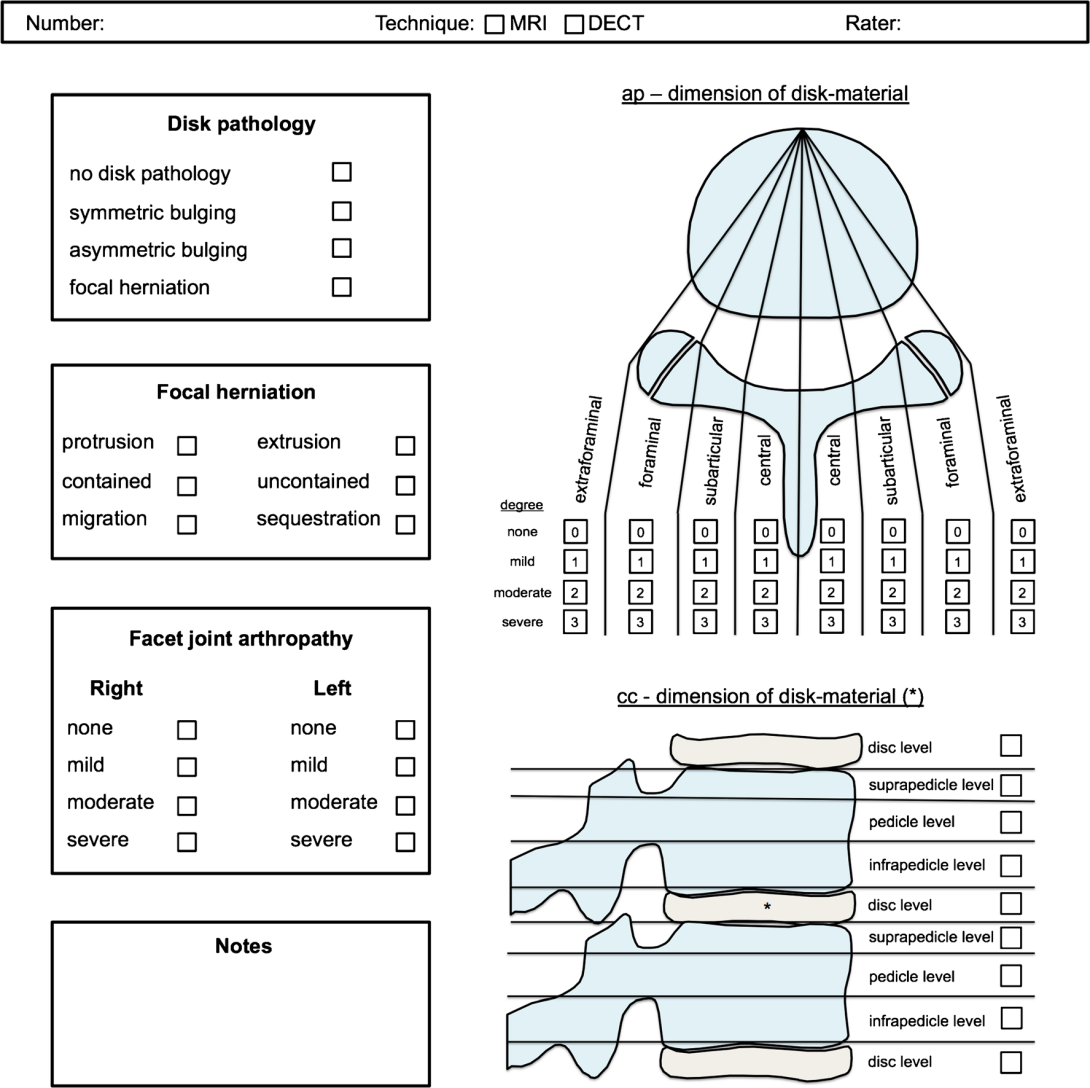
Scoring sheet. Granular scoring system for the assessment of disks and facet joints. *AP* anteroposterior, *CC* craniocaudal

For the assessment of disks, a granular scoring system was developed including both current classification and morphology (Fig. 2). Specifically, the system includes a 4-point scale for assessing general disk pathology (“no disk pathology,” “symmetric bulging,” “asymmetric bulging,” or “focal herniation”) and, if focal herniation is present, a subscale for assessing the type (protrusion or extrusion and migration or sequestration) as recently proposed [11]. Furthermore, craniocaudal and anteroposterior displacement of disk material by anatomical region was assessed using a 4-point semiquantitative grading system (0: no compromise, 1: mild compromise of less than one-third, 2: moderate compromise between one- and two-thirds, and 3: severe compromise of more than two-thirds of the corresponding anatomical region). The following anatomical regions were scored: central, subarticular, foraminal, and extraforaminal for anteroposterior disk displacement from both sides from midline and infrapedicular, pedicular, suprapedicular, and diskal for craniocaudal disk displacement in both directions from the target/reference disk.

### Data postprocessing

For analysis of the degree of anteroposterior disk displacement, a sum score of all single scores for the eight anatomical regions was calculated. Thus, the sum score of all anatomical regions ranged from 0 to 24.

For the calculation of diagnostic accuracy, the scales “general disk pathology” and “craniocaudal disk displacement” were dichotomized as follows: 0: no disk pathology, 1: disk pathology and 0: no disk migration, 1: disk migration, respectively.

A two-out-of-three-reader agreement approach was used for all scored parameters with the exception of anteroposterior sum scores (mean as group value). Further disagreements were solved in consensus.

### Statistical analysis

For determination of interrater reliability between the three readers for each imaging technique (MRI, CT, and DECT) or between reader group values (MRI vs. DECT, MRI vs. CT) for the assessment of intermodality agreement, a two-way random single-measure (or average measure for anteroposterior sum score group values) intraclass correlation coefficient (ICC) was calculated for the following variables: general disk pathology (semiquantitative score), craniocaudal disk displacement (semiquantitative score), and anteroposterior disk displacement (sum scores and separately for each anatomical region with semiquantitative scores). For analysis of correlation or differences between MRI and DECT and between MRI and CT sum score mean values of anteroposterior disk displacement, a paired *t* test was calculated.

For the dichotomized variables, contingency table analysis was performed with MRI as standard of reference. For the dichotomized variables “general disk pathology” and “craniocaudal disk migration,” we additionally calculated Cohen’s kappa to determine the agreement between DECT and MRI and between CT and MRI. The statistical significance level for all tests performed was α = 0.05, and statistical analysis was performed using R (Version 3.6.3) with R-Studio (Version 1.1.463).

## Results

### Patients

A total of 25 patients who underwent DECT-guided lumbar spine injections were identified, and 13 patients were included in our retrospective analysis (7 women and 6 men; mean age, 57.9 ± 18.7 years). The distribution of the 13 pathologic target levels (one per patient) was as follows: 8x L4/L5, 3x L3/L4, and 2x L5/S1. In eight patients, we were able to additionally define a normal-appearing reference level: 3x L3/L4, 2x L5/S1, 1x Th12/L1, L1/L2, and L2/L3. Five patients had degenerative changes of all scanned lumbar spine segments, and no reference level could be defined. The mean CTDIvol (computed tomography dose index) of DECT was 13.07 ± 10.64 mGy. The mean DLP (dose-length product) was 252.8 ± 186.24 mGy*cm (see Table 1 for details).

**Table 1:** Patient characteristics

No.	Age	Sex	Target	Reference	DECT scan volume	CTDI	DLP	MRI sum score	DECT sum score	CT sum score
1	57	f	L4 – L5	L3 – L4	L3 – S2	45.70	755.30	8.00	8.67	6.00
2	63	m	L3 – L4	L1 – L2	L1 – S2	15.70	397.80	6.67	14.00	7.67
3	38	f	L4 – L5	L3 – L4	L2 – L5	6.00	79.10	7.00	6.00	5.67
4	89	m	L4 – L5	T12 – L1	Th11 – L5	10.27	260.70	20.00	20.33	10.00
5	38	m	L5 – S1	L3 – L4	L3 – S1	10.10	312.60	4.00	2.33	4.67
6	50	f	L4 – L5	L5 – S1	L4 – S1	5.20	60.50	4.00	1.00	7.00
7	40	f	L4 – L5	L2 – L3	L1 – S3	6.28	164.60	10.67	11.67	12.33
8	72	f	L3 – L4	L5 – S1	L1 – S4	11.03	282.40	9.33	7.33	7.33
9	77	m	L5 – S1	–	L4 – S4	12.20	224.30	9.67	7.67	9.00
10	70	f	L4 – L5	–	L3 – S3	9.80	156.60	10.67	13.33	9.67
11	42	m	L4 – L5	–	L4 – S1	11.20	147.30	9.33	9.00	4.00
12	81	f	L3 – L4	–	L2 – S2	20.00	364.30	9.00	9.00	6.33
13	35	m	L4 – L5	–	L3 – L5	6.40	80.70	7.00	6.00	4.00

No.: patient number; age: patient age at time of DECT examination; sex: male/female; target: level of target disks/facets; reference: level of reference disks/facets; DECT scan volume: area scanned by DECT; CTDI: computed tomography dose index in mGy; DLP: dose-length product in mGy*cm; MRI sum score: average sum score for anteroposterior target disk displacement on semiquantitative scale in MRI; DECT sum score: average sum score for anteroposterior target disk displacement on semiquantitative scale in DECT, CT sum score: average sum score for anteroposterior target disk displacement on semiquantitative scale in CT

### Scoring

**Fig. 3:**
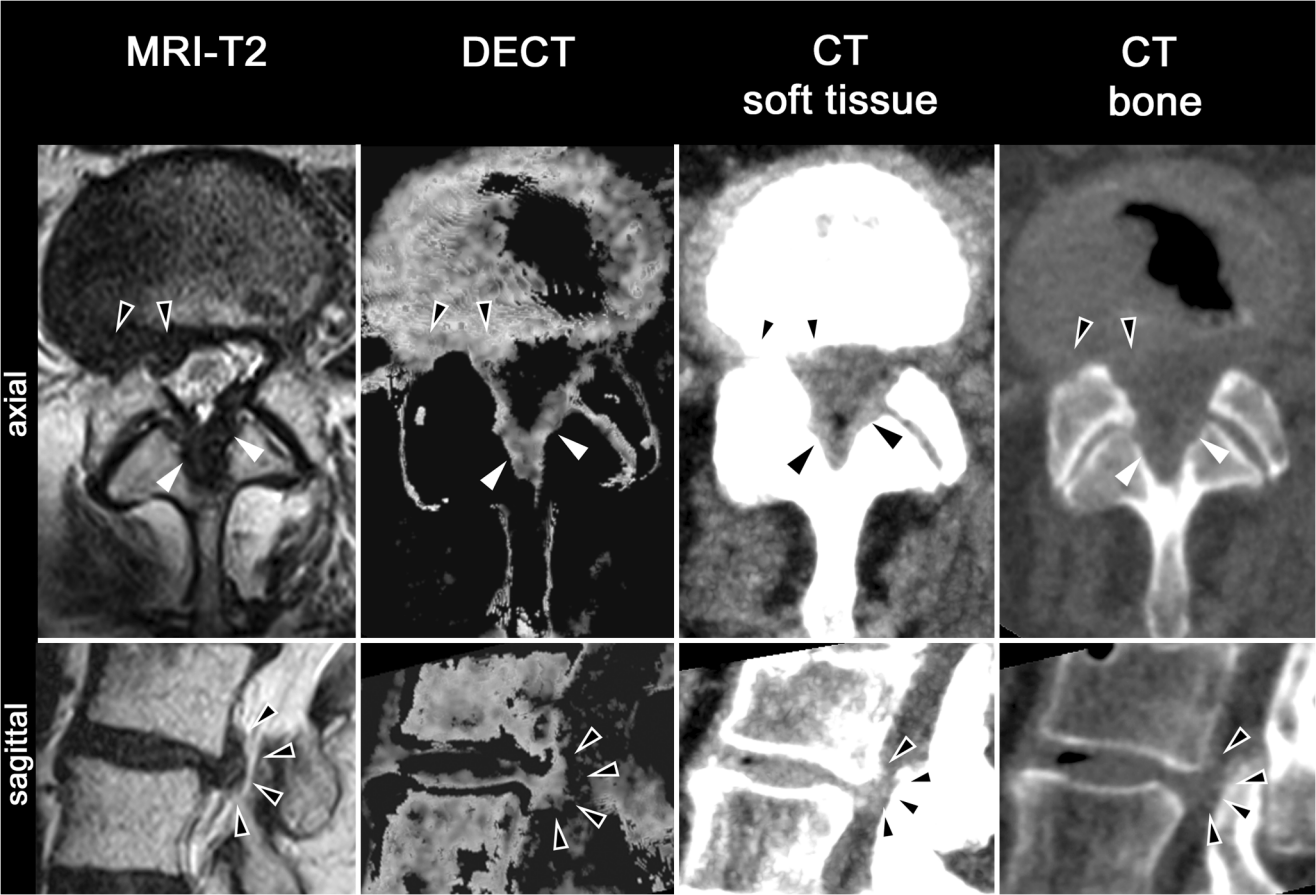
Representative example of a scored imaging dataset. Axial and sagittal T2w MR images, collagen-reconstructed DECT images, and conventional (DE-)CT images in soft tissue and bone window of a patient with focal herniation with extrusion and disk migration into right subarticular, foraminal, and extraforaminal areas. Arrowheads: displaced disk material; filled arrowheads: ligamentum flavum

A representative example of a scored imaging dataset is shown in Fig. 3. T2w MR images and corresponding collagen-reconstructed DECT images for all target levels and for one reference level are provided in Supplement 1.

#### General disk pathology

In the 21 cases analyzed (13 target and 8 reference disks in 13 patients), DECT was positive for disk pathology in 13 cases, CT in 14 cases, and MRI in 15 cases. Results of the contingency table analysis for DECT and CT are shown in Table 2. For DECT, this analysis yielded a specificity of 1.00 (0.54–1.00) and a sensitivity of 0.87 (0.60–0.98), while CT reached a specificity of 1.00 (0.54–1.00) and a sensitivity of 0.93 (0.68–1.00). Cohen’s kappa calculated with the dichotomized scores for agreement between DECT and MRI was 0.788 (*p* < 0.001) and 0.889 (*p* < 0.001) for the agreement between CT and MRI.

**Table 2:** Contingency analysis of general disk pathology—DECT/CT

General disk pathology	MRI+	MRI−	Total	SE	0.87	0.60 to 0.98	General disk pathology	MRI+	MRI−	Total	SE	0.93	0.68 to 1.00
DECT+	13	0	13	SP	1.00	0.54 to 1.00	CT +	14	0	14	SP	1.00	0.54 to 1.00
DECT-	2	6	8	PPV	1.00	0.75 to 1.00	CT-	1	6	7	PPV	1.00	0.77 to 1.00
Total	15	6	21	NPV	0.75	0.35 to 0.97	Total	15	6	21	NPV	0.86	0.42 to 1.00

*SE* sensitivity, *SP* specificity, *PPV* positive predictive value, *NPV* negative predictive value. Data are given with 95% confidence intervals. All values were calculated based on agreement of at least two of the three readers using MRI as standard of reference

The results for interrater/intermodality agreement for the semiquantitative scores are summarized in Supplement 2. ICCs for interrater agreement were 0.649 (0.42–0.823) for DECT, 0.319 (0.061–0.597) for CT, and 0.493 (0.228–0.727) for MRI. ICCs for the agreement between the modalities was 0.321 (− 0.136–0.659) between DECT and MRI and 0.521 (0.115–0.775) between CT and MRI.

#### Anteroposterior disk displacement

**Fig. 4:**
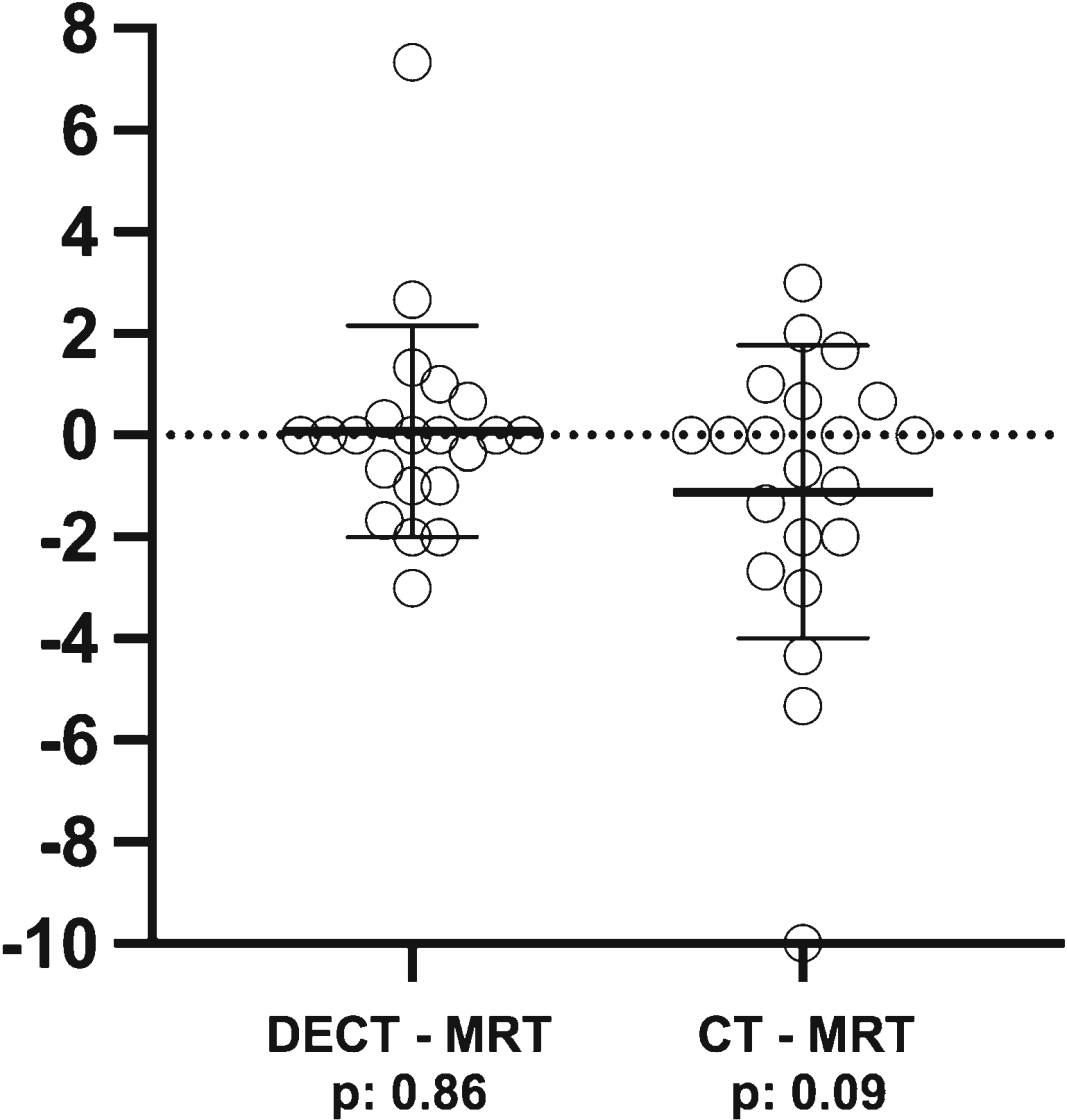
Correlation analysis of the anteroposterior degree of disk displacement between MRI and DECT. DECT and CT values for anteroposterior disk displacement after subtraction of corresponding MRI data points with mean and standard deviation. *p* values calculated by a paired *t* test between MRI and DECT and between MRI and CT data points of anteroposterior disk displacement

The mean sum score for anteroposterior disk displacement in all patients was 5.97 ± 5.64 for DECT, 4.78 ± 3.83 for CT, and 5.88 ± 5.09 for MRI. Intermodality analysis performed with a paired *t* test revealed *p* values of 0.86 for differences between DECT and MRI and 0.09 between CT and MRI. Figure 4 illustrates all DECT and CT sum scores for anteroposterior disk displacement after subtraction of the corresponding MRI sum scores, resulting in a mean value of 0.08 (SD: 2.08) for DECT and − 1.11 (SD: 2.88) for CT.

Analysis of interrater agreement revealed an ICC of 0.82 (0.666–0.916) for DECT, 0.624 (0.39–0.808) for CT, and 0.848 (0.71–0.93) for MRI. Between-modality ICC was 0.963 (0.909–0.985) for interrater agreement between DECT and MRI and 0.876 (0.691–0.95) for agreement between CT and MRI (Table 3).

**Table 3:** Interrater/intermodality agreement for anteroposterior degree of disk displacement (semiquantitative sum scores)

	ICC	*p*	95% CI
Interrater agreement MRI (R1 vs. R2 vs. R3)	0.848	< 0.001	0.71 < ICC < 0.93
Interrater agreement DECT (R1 vs. R2 vs. R3)	0.82	< 0.001	0.666 < ICC < 0.916
Interrater agreement CT (R1 vs. R2 vs. R3)	0.624	< 0.001	0.39 < ICC < 0.808
Interrater agreement MRI vs. DECT (mean vs. mean)	0.963	< 0.001	0.909 < ICC < 0.985
Interrater agreement MRI vs. CT (mean vs. mean)	0.876	< 0.001	0.691 < ICC < 0.95

R1, R2, R2: reader one, two, and three; mean: mean score of the three readers’ individual sum scores; ICC: intraclass correlation coefficient; p: probability of ICC-linked F-statistic; 95% CI: 95% confidence interval for ICC values

ICCs calculated by anatomical region are presented in Supplement 3a and 3b.

#### Craniocaudal disk displacement

Seven cases showed disk migration in DECT, five in CT, and six in MRI. Results of the contingency table analysis for DECT and CT are summarized in Table 4. DECT showed a specificity of 0.80 (0.54–1.00) and a sensitivity of 0.67 (0.22–0.96), while CT showed a specificity of 0.93 (0.68–1.00) and a sensitivity of 0.67 (0.22–0.96). Cohen’s kappa calculated with the dichotomized scores was 0.444 (*p* = 0.04) for the agreement between DECT and MRI and 0.632 (*p* = 0.004) for the agreement between CT and MRI.

**Table 4:** Contingency analysis of disk migration—DECT/CT

Disk migration	MRI+	MRI−	Total	SE	0.67	0.22 to 0.96	Disk migration	MRI+	MRI−	Total	SE	0.67	0.22 to 0.96
DECT+	4	3	7	SP	0.80	0.54 to 1.00	CT+	4	1	5	SP	0.93	0.68 to 1.00
DECT−	2	12	14	PPV	0.57	0.18 to 0.90	CT−	2	14	16	PPV	0.80	0.28 to 0.99
Total	6	15	21	NPV	0.86	0.57 to 0.98	Total	6	15	21	NPV	0.88	0.62 to 0.98

*SE* sensitivity, *SP* specificity, *PPV* positive predictive value, *NPV* negative predictive value. Data are given with 95% confidence intervals. All values were calculated based on agreement of at least two of the three readers using MRI as standard of reference

The results for interrater agreement for the semiquantitative scores are summarized in Supplement 4. ICCs for interrater agreement were 0.602 (0.356–0.796) for DECT, 0.289 (0.033–0.571) for CT, and 0.483 (0.224–0.718) for MRI. ICCs for between-modality agreement was 0.63 (0.285–0.831) for DECT and MRI and 0.489 (0.107–0.751) for CT and MRI.

## Discussion

This is the first study investigating the diagnostic accuracy of collagen-sensitive mapping using DECT in the detection of disk pathology in comparison with MRI as the current diagnostic standard and with conventional CT. Our results show high diagnostic accuracy for both DECT (0.87 sensitivity and 1.00 specificity) and conventional CT (0.93 sensitivity and 1.00 specificity) in determining whether disk pathology is present using MRI as standard of reference. However, DECT had remarkably higher values for within-modality interrater agreement than CT for semiquantitative scoring (ICC: 0.649 vs. 0.319) regarding the presence of general disk pathology, indicating greater interindividual consistency of scoring.

While readers agreed regarding the presence of a disk pathology, they disagreed regarding the kind of pathology despite using an established classification system (ICCs: 0.493 (MRI), 0.649 (DECT), 0.319 (CT)). This observation underscores uncertainties known from clinical practice in terms of handling a complex and changing nomenclature. Thus, our finding calls into question the practicability of the established system for reliably classifying disk pathologies especially since the classification’s impact on treatment remains unclear. The abovementioned tendency towards higher interrater agreement in the interpretation of DECT compared to CT was also found for the semiquantitative values of the anteroposterior (ICC: 0.82 vs. 0.624) as well as craniocaudal disk displacement (ICC: 0.602 vs. 0.289).

Analysis of the sum scores for anteroposterior disk displacement shows a tendency towards underestimation of disk pathology based on CT (mean: − 1.11) compared with DECT (mean 0.08) using MRI as reference. Consistently, the ICC for the agreement between DECT and MRI was excellent as well (0.963) and higher than for the agreement between CT and MRI (0.876).

The slight to moderate decreases in ICCs in the separate testing for agreement of scores for anteroposterior disk displacement per anatomical location may be explained by the small sample size of this retrospective analysis and differences among the readers in exactly assigning propagated disk material to neighboring scoring spaces. Even though the clinical relevance of classifying disk pathologies is rather low for patients suffering from radiculopathy, clinicoradiographic correlation could identify certain morphological pathologies of greater clinical relevance.

The observed tendency towards better correlation between DECT and MRI in the assessment of the degree of anteroposterior disk displacement and the superior intermodality agreement with MRI suggest that DECT may provide valuable additional information for the evaluation of disk pathology and improve diagnostic reliability whenever a CT is performed. That may be the case in patients with contraindications to MRI or who undergo a CT scan for other indications (e.g., in an emergency setting). Here, it is important to note that when generating DECT images, conventional CT images are always acquired as well. Thus, DECT should not be regarded as a separate imaging test but rather as a supplement providing useful additional information in patients undergoing conventional CT.

To date, DECT has predominantly been investigated for its ability to detect malignant bone marrow infiltration, posttraumatic bone marrow lesions, or traumatic intervertebral disk injuries [6, 12–14]. Especially in patients with vertebral compression fractures, several studies have underlined the added value of DECT compared with conventional CT imaging [4, 5, 7]. In patients with vertebral fractures, Pumberger et al. showed high sensitivity (0.85) and specificity (0.75) of collagen-sensitive DECT in detecting intervertebral disk injuries and higher interrater reliability compared with MRI. In conjunction with our findings, these results suggest that DECT may aid in the diagnosis of disk pathologies in different settings in the future.

A previous study by Booz et al. showed higher sensitivity and specificity of DECT compared with standard CT in detecting lumbar disk herniation and spinal nerve root impingement, suggesting that DECT might be as accurate as MRI [8]. However, Booz et al. used a different reconstruction algorithm (virtual non-calcium reconstruction), while we investigated a three-material decomposition algorithm with a collagen-specific gradient.

Some limitations have to be discussed. Our analysis is limited by the small sample size of 13 patients and retrospective data collection. Therefore, the statistical analysis may be underpowered, and we found only tendencies but no statistically significant differences between CT and DECT. Hence, our results need to be validated in a larger prospective setting. Direct measurement of diagnostic confidence should also be added. Furthermore, we only included patients with known disk pathologies. Therefore, we analyzed other, normal-appearing, lumbar segments in the same patients to serve as an artificial control group. However, we took great effort to blind the readers to whether a pathological or healthy disk was scored by only showing individual spinal levels. The results cannot be transferred to the cervical spine as this was beyond the scope of our study.

In conclusion, our study provides initial evidence of a high diagnostic accuracy of collagen-specific mapping based on DECT scans in depicting vertebral morphology and assessing lumbar disk pathologies, allowing more detailed and reliable assessment compared with conventional CT. This technique might be used to obtain additional information whenever a spinal CT scan is performed, e.g., for trauma imaging or a CT-guided intervention. However, before DECT can be introduced as a valid alternative in daily clinical routine, further studies in more patients need to be performed.

## Supplementary information

ESM 1 **Supplement 1 T2w MR images and collagen-reconstructed DECT images of all target levels and one representative reference level.** Arrowheads: displaced disk material; filled arrowheads: ligamentum flavum. (PNG 7525 kb)

High Resolution Image (TIF 44863 kb)

ESM 2 (DOCX 20 kb)

ESM 3 (DOCX 26 kb)

ESM 4 (DOCX 20 kb)
