# Supplementary figures and images for: Vertebral disk morphology of the lumbar spine: a retrospective analysis of collagen-sensitive mapping using dual-energy computed tomography
Source: Skeletal Radiol. 2020 Dec 4;50(7):1359–67. doi: 10.1007/s00256-020-03685-5 (PMC8119261; doi:10.1007/s00256-020-03685-5)

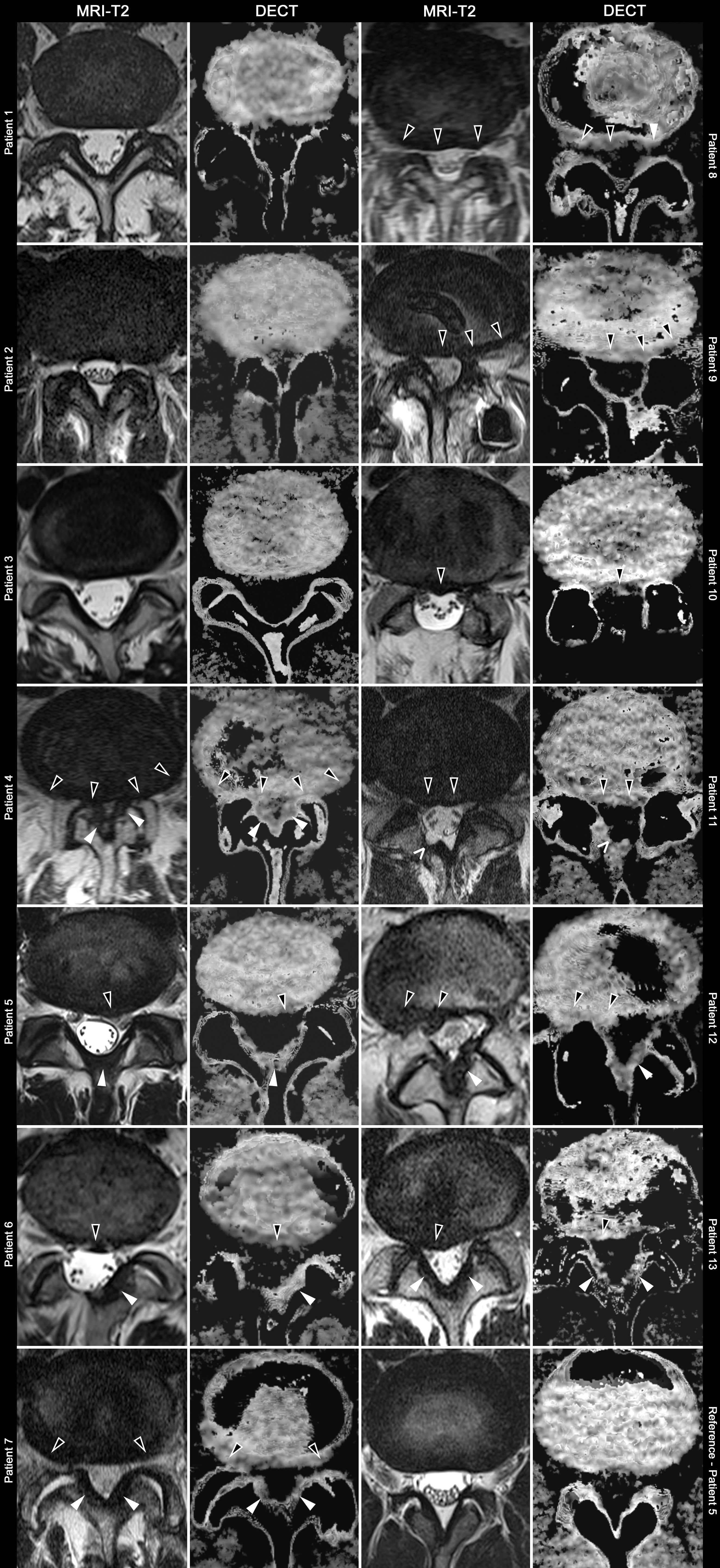

Supplement: Supplementary file 1 — Supplement 1 T2w MR images and collagen-reconstructed DECT images of all target levels and one representative reference level. Arrowheads: displaced disk material; filled arrowheads: ligamentum flavum. (PNG 7525 kb) [file 256_2020_3685_Fig5_ESM.png]

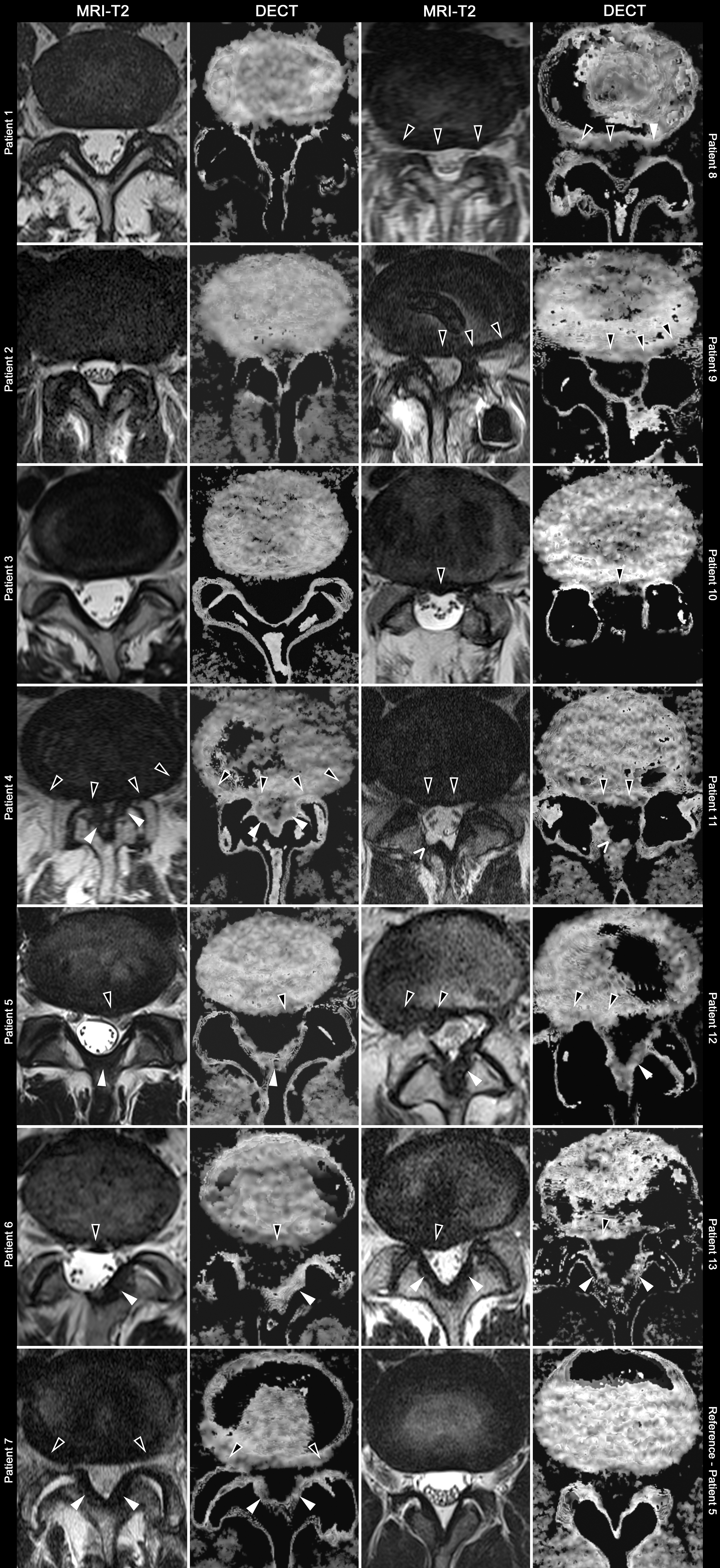

Supplement: Supplementary file 2 — High Resolution Image (TIF 44863 kb) [file 256_2020_3685_MOESM1_ESM.tif]
