# Supplementary material for: Vertebral disk morphology of the lumbar spine: a retrospective analysis of collagen-sensitive mapping using dual-energy computed tomography
Source: Skeletal Radiol. 2020 Dec 4;50(7):1359–67. doi: 10.1007/s00256-020-03685-5 (PMC8119261; doi:10.1007/s00256-020-03685-5)
Supplement: Supplementary file 3 — (DOCX 20 kb) [file 256_2020_3685_MOESM2_ESM.docx]

|  | ICC | p | 95% CI |
| --- | --- | --- | --- |
| **Interrater Agreement MRI**  (R1 vs. R2 vs. R3) | 0.493 | < 0.001 | 0.228 < ICC < 0.727 |
| **Interrater Agreement DECT**  (R1 vs. R2 vs. R3) | 0.649 | < 0.001 | 0.420 < ICC < 0.823 |
| **Interrater Agreement CT**  (R1 vs. R2 vs. R3) | 0.319 | 0.007 | 0.061 < ICC < 0.597 |
| **Intermodality Agreement MRI vs. DECT**  (Consensus vs. Consensus) | 0.321 | 0.08 | -0.136 < ICC < 0.659 |
| **Intermodality Agreement MRI vs. CT**  (Consensus vs. Consensus) | 0.521 | 0.008 | 0.115 < ICC < 0.775 |

**Supplement 2 Interrater/-modality agreement for presence of general disk pathology (semiquantitative score).** R1, R2, R2: reader one, two, and three; Consensus: consensus score of the three readers’ individual scores; ICC: intraclass correlation coefficient; p: probability of ICC-linked F-statistic; 95% CI: 95% confidence interval for ICC values.
