# Supplementary material for: Vertebral disk morphology of the lumbar spine: a retrospective analysis of collagen-sensitive mapping using dual-energy computed tomography
Source: Skeletal Radiol. 2020 Dec 4;50(7):1359–67. doi: 10.1007/s00256-020-03685-5 (PMC8119261; doi:10.1007/s00256-020-03685-5)
Supplement: Supplementary file 4 — (DOCX 26 kb) [file 256_2020_3685_MOESM3_ESM.docx]

|  | ICC | p | 95% CI | ICC | p | 95% CI |
| --- | --- | --- | --- | --- | --- | --- |
|  | **Right extraforaminal** | | | **Left extraforaminal** | | |
| **Interrater Agreement MRI**  (R1 vs. R2 vs. R3) | 0.679 | < 0.001 | 0.462 < ICC < 0.84 | 0.495 | < 0.001 | 0.238 < ICC < 0.725 |
| **Interrater Agreement DECT**  (R1 vs. R2 vs. R3) | 0.544 | < 0.001 | 0.293 < ICC < 0.758 | 0.697 | < 0.001 | 0.487 < ICC < 0.85 |
| **Interrater Agreement CT**  (R1 vs. R2 vs. R3) | 0.379 | 0.003 | 0.104 < ICC < 0.648 | 0.383 | 0.003 | 0.108 < ICC < 0.651 |
| **Interrater Agreement MRI vs. DECT**  (Consensus vs. Consensus) | 0.785 | < 0.001 | 0.542 < ICC < 0.907 | 0.483 | 0.013 | 0.065 < ICC < 0.754 |
| **Interrater Agreement MRI vs. CT**  (Consensus vs. Consensus) | 0.63 | 0.002 | 0.246 < ICC < 0.836 | 0.512 | 0.005 | 0.135 < ICC < 0.765 |

**fghfg**

|  | **Right foraminal** | | | **Left foraminal** | | |
| --- | --- | --- | --- | --- | --- | --- |
| **Interrater Agreement MRI**  (R1 vs. R2 vs. R3) | 0.826 | < 0.001 | 0.68 < ICC < 0.919 | 0.588 | < 0.001 | 0.333 < ICC < 0.789 |
| **Interrater Agreement DECT**  (R1 vs. R2 vs. R3) | 0.62 | < 0.001 | 0.368 < ICC < 0.809 | 0.51 | < 0.001 | 0.256 < ICC < 0.735 |
| **Interrater Agreement CT**  (R1 vs. R2 vs. R3) | 0.443 | < 0.001 | 0.177 < ICC < 0.691 | 0.523 | < 0.001 | 0.266 < ICC < 0.745 |
| **Interrater Agreement MRI vs. DECT**  (Consensus vs. Consensus) | 0.847 | < 0.001 | 0.666 < ICC < 0.935 | 0.757 | < 0.001 | 0.487 < ICC < 0.894 |
| **Interrater Agreement MRI vs. CT**  (Consensus vs. Consensus) | 0.576 | 0.002 | 0.218 < ICC < 0.801 | 0.558 | 0.002 | 0.194 < ICC < 0.791 |

ghj

|  | **Right subarticular** | | | **Left subarticular** | | |
| --- | --- | --- | --- | --- | --- | --- |
| **Interrater Agreement MRI**  (R1 vs. R2 vs. R3) | 0.832 | < 0.001 | 0.691 < ICC < 0.921 | 0.696 | < 0.001 | 0.481 < ICC < 0.85 |
| **Interrater Agreement DECT**  (R1 vs. R2 vs. R3) | 0.567 | < 0.001 | 0.318 < ICC < 0.774 | 0.518 | < 0.001 | 0.258 < ICC < 0.743 |
| **Interrater Agreement CT**  (R1 vs. R2 vs. R3) | 0.543 | < 0.001 | 0.286 < ICC < 0.759 | 0.531 | < 0.001 | 0.278 < ICC < 0.75 |
| **Interrater Agreement MRI vs. DECT**  (Consensus vs. Consensus) | 0.855 | < 0.001 | 0.677 < ICC < 0.939 | 0.568 | 0.003 | 0.198 < ICC < 0.798 |
| **Interrater Agreement MRI vs. CT**  (Consensus vs. Consensus) | 0.679 | < 0.001 | 0.363 < ICC < 0.856 | 0.71 | < 0.001 | 0.413 < ICC < 0.871 |

h

|  | **Right central** | | | **Left central** | | |
| --- | --- | --- | --- | --- | --- | --- |
| **Interrater Agreement MRI**  (R1 vs. R2 vs. R3) | 0.659 | < 0.001 | 0.435 < ICC < 0.829 | 0.581 | < 0.001 | 0.336 < ICC < 0.782 |
| **Interrater Agreement DECT**  (R1 vs. R2 vs. R3) | 0.689 | < 0.001 | 0.476 < ICC < 0.845 | 0.617 | < 0.001 | 0.376 < ICC < 0.805 |
| **Interrater Agreement CT**  (R1 vs. R2 vs. R3) | 0.372 | 0.003 | 0.101 < ICC < 0.641 | 0.392 | 0.001 | 0.132 < ICC < 0.652 |
| **Interrater Agreement MRI vs. DECT**  (Consensus vs. Consensus) | 0.875 | < 0.001 | 0.717 < ICC < 0.947 | 0.704 | < 0.001 | 0.41 < ICC < 0.867 |
| **Interrater Agreement MRI vs. CT**  (Consensus vs. Consensus) | 0.778 | < 0.001 | 0.53 < ICC < 0.903 | 0.84 | < 0.001 | 0.65 < ICC < 0.932 |

l

**Supplement 3 Interrater agreement for anteroposterior degree of disk displacement (semiquantitative score).** R1, R2, R2: reader one, two and three; Consensus: consensus score of the three readers’ individual scores; ICC: intraclass correlation coefficient; p: probability of ICC-linked F-statistic; 95% CI: 95% confidence interval for ICC values.
